# The Effect of *Bifidobacterium animalis* subsp. *lactis* Bl-04 on Influenza A Virus Infection in Mice

**DOI:** 10.3390/microorganisms11102582

**Published:** 2023-10-17

**Authors:** Bryan Zabel, Sanna M. Mäkelä, Derek Nedveck, Ashley A. Hibberd, Nicolas Yeung, Sinikka Latvala, Liisa Lehtoranta, Jouni Junnila, Kevin B. Walters, Wesley Morovic, Markus J. Lehtinen

**Affiliations:** 1 Health & Biosciences, International Flavors & Fragrances, 3329 Agriculture Dr., Madison, WI 53716, USA; 2 Health & Biosciences, International Flavors & Fragrances, Sokeritehtaantie 20, 02460 Kantvik, Finland; 3 4Pharma Ltd., Tykistökatu 4D, 20520 Turku, Finland; jouni.junnila@estimates.fi; 4 Department of Infectious Disease Research, Southern Research Institute, 431 Aviation Way, Frederick, MD 21701, USA

**Keywords:** probiotic, influenza, immune response, animal study, *Bifidobacterium*, transcriptomics

## Abstract

Influenza A virus infection is a major global disease requiring annual vaccination. Clinical studies indicate that certain probiotics may support immune function against influenza and other respiratory viruses, but direct molecular evidence is scarce. Here, mice were treated with a placebo or *Bifidobacterium animalis* subsp. *lactis* Bl-04 (Bl-04) orally via food (cereal) and also by gavage and exposed to Influenza A virus H1N1 (H1N1). The symptoms of the infection were observed, and tissues and digesta were collected for viral load RT-qPCR, transcriptomics, and microbiomics. The treatment decreased the viral load by 48% at day 3 post-infection in lungs and symptoms of infection at day 4 compared to placebo. Tissue transcriptomics showed differences between the Bl-04 and placebo groups in the genes in the Influenza A pathway in the intestine, blood, and lungs prior to and post-infection, but the results were inconclusive. Moreover, 16S rRNA gene profiling and qPCR showed the presence of Bl-04 in the intestine, but without major shifts in the microbiome. In conclusion, Bl-04 treatment may influence the host response against H1N1 in a murine challenge model; however, further studies are required to elucidate the mechanism of action.

## 1. Introduction

It is estimated that the seasonal influenza viruses infect about one billion humans annually, causing three to five million severe cases and over 500,000 deaths [1,2]. It is not fully known why some individuals suffer from severe respiratory disease and some are asymptomatic, but age, comorbidities such as diabetes, lung or cardiac diseases, obesity, immunocompromising conditions, genetics, and microbiota composition are known to increase the risk of severe illness [3,4,5]. Studies of the respiratory microbiome during Influenza A virus (IAV, alphainfluenzavirus influenzae) infection show that a lower viral susceptibility correlates with the abundance of some organisms like *Prevotella* spp. [3,4]. Mechanistic studies show that an intact commensal microbiota provides the first signal needed for inflammasome activation during IAV infection, thus leading to more effective Th1, cytotoxic T lymphocyte (CTL), and IgA responses [6]. Microbes can also regulate the adaptive immune response, such as priming of CD8+ and CD4+ T-cells or antibody production [6,7].

Intestinal microbes communicate with the lung via the gut–lung axis, and the link between the gut microbiota and respiratory infections [8] has led to investigations of probiotics and their possible effects on viral respiratory infections. Meta-analyses conducted on the effects of probiotics on acute upper respiratory infection have suggested that they reduce the incidence of infection and the severity of symptoms [9,10,11]. However, these effects are specific to the probiotic strain used, as suggested by the variability in the efficacy results in meta-analyses [9,12]. Thus, more mechanistic studies of specific probiotic strains are needed to understand how the probiotic or corresponding alterations in the microbiota composition may elicit or correlate to host immune responses. *Bifidobacterium animalis* subsp*. lactis* Bl-04 (Bl-04) has been shown to reduce the risk of upper respiratory illness in healthy adults [13], but no significant changes were detected in immune markers pre- to post-supplementation [13,14]. Bl-04 was further shown to decrease [15] or have no impact on [16] rhinovirus load in nasal washes obtained in a rhinovirus challenge study. In these studies, higher interleukin (IL)-8 or IL-1β levels were measured in nasal washes post-supplementation, but prior to infection, suggesting there was a priming effect of the probiotic on the immune system [16,17]. However, the mechanistic action of Bl-04 on anti-viral immune function remains elusive.

Mice are used extensively as a model for IAV infection, although they do not fully recapitulate IAV infection in humans [18]. Some human influenza viruses do not cause disease in mice, necessitating the use of mouse-adapted viral strains in experiments. However, the 2009 pandemic H1N1 influenza virus readily infects mice and results in the manifestation of symptoms such as anorexia, huddling, hunching, fur ruffling, dehydration, and hypothermia [19,20]. Furthermore, both the innate and adaptive immune responses to IAV infection have been measured in mice using next-generation sequencing (NGS) methods. For example, previous research that measured murine gene expression by RNA-sequencing (RNA-seq) after influenza virus challenge showed a tissue-specific innate immune reaction, specifically initiation by natural killer (NK) cell gene groups, as well as B-cell and T-cell signature gene activation [21].

While probiotics in general [9,10,11,12] and Bl-04 specifically [13] have shown efficacy against viral respiratory infections, the evidence for the mechanism of action of probiotics is lacking. We hypothesized that the treatment of mice with Bl-04 prior to and post Influenza A/California/07/2009 (H1N1) virus infection could modulate the microbiota and the innate immune system of the mice to reduce viral load and associated symptoms. We applied transcriptomics to assess the host immune response and 16S rRNA gene sequencing to analyze the effects on the microbiota.

## 2. Materials and Methods

### 2.1. Ethics Statement

The study design was reviewed by the Institutional Animal Care and Use Committee (IACUC) (No. 17-01-003B) and Institutional Biosafety Committee (IBC) (No. R18-03-004SA) at Southern Research. Southern Research is fully accredited by the Association for Assessment and Accreditation of Laboratory Animal Care (AAALAC) International. The authors complied with the ARRIVE guidelines. All methods were performed in accordance with relevant guidelines and regulations.

### 2.2. Mice and Infection Model

Female BALB/c mice were obtained from Jackson Laboratories and group-housed at the Southern Research facility (Frederick, MD, USA) with a 12/12 h light/dark cycle with food and water available ad libitum. The mice were acclimatized and quarantined for 6 days before the experiments and monitored twice daily throughout the study. Six- to seven-week-old mice (*n* = 106) were randomized using Provantis (Instem™ LSS Ltd., Staffordshire, UK) into two groups based on weight and received either probiotic or placebo orally via food (cereal) and gavage for 21 days prior to and for 13 days post-intranasal infection with a sublethal dose of influenza virus strain A/California/07/2009 (Figure S1). The probiotic strain administered to the mice was *B. animalis* subsp*. lactis* Bl-04 (Danisco Cultures, Madison, AL, USA). A freshly prepared probiotic at a 1 × 10^9^ colony forming unit (CFU) dose in 0.2 mL water was administered by gavage and another 1 × 10^9^ CFU dose by addition to a cereal flake that was fed to each mouse daily. The total dose of probiotic was 2 × 10^9^ CFU/day with maltodextrin as a vehicle. Maltodextrin alone was used as a placebo at a dose of 0.1 g by gavage and 0.1 g in one cereal flake per day. The influenza virus strain A/California/07/2009 (H1N1) was propagated in eggs and the titer was determined using a plaque-forming assay. The challenge inoculum 5 × 10^3^ PFU/animal was diluted into DPBS (Gibco, Thermo Fisher Scientific, Waltham, MA, USA) and was intranasally administered in 100 µL volume, 50 µL per naris per mouse in both groups.

All mice were monitored twice daily for signs of morbidity and mortality. Daily body weights and clinical observations such as rough coat, hunched posture, and abnormal breathing were recorded starting on day 0, prior to challenge. On days −21, −20, −18, −14, −7, 0, 3, 5, and 7, a subset of mice was euthanized, and tissues and blood were collected. All remaining mice were euthanized on day 14 with no tissues or blood collected. Viral load was measured using reverse transcription quantitative real-time PCR (RT-qPCR) on post-challenge samples (days 3, 5, and 7). Anesthesia for viral challenge was carried out using a ketamine 12.5 mg/mL/xylazine 2.5 mg/mL cocktail, intraperitoneally, at 0.05–0.1 mL/mouse. The mice were euthanized by intraperitoneal barbiturate overdose. Animals that did not have lungs collected were euthanized via asphyxiation with CO_2_. Blood samples were collected via cardiac puncture. Tissue from the lungs, duodenum, jejunum, ileum, and mediastinal and mesenteric lymph nodes were collected for viral load and transcriptomic analyses and digesta for microbiota analysis (Figure S2).

### 2.3. Viral Load Analysis

The viral loads in the lung tissue were measured by the detection of pandemic H1N1 influenza virus RNA using RT-qPCR. Lung tissue was homogenized in PBS using a bead mill (Omni International, Kennesaw, GA, USA). Viral RNA was isolated from the homogenate using the QIAamp Viral RNA mini kit (Qiagen, Hilden, Germany) following the manufacturer’s instructions. An RNA standard was produced by in vitro transcription and quantified using a nanodrop spectrophotometer. Serially diluted, quantified RNA standards were used to generate a standard curve that allowed for the absolute quantitation of viral RNA in each biological sample. All RT-qPCR reactions were performed using the Taqman fast virus 4× Master Mix (Life Technologies, Carlsbad, CA, USA) and were run in QuantStudios 6 using the standard cycle. Viral load is expressed as viral RNA copies per mg of extracted lung tissue.

### 2.4. Microbial DNA Extraction and Quantitative Real-Time PCR (qPCR)

Mouse intestine was sectioned and digesta was pushed out of duodenum, jejunum, ileum, and caecum (Figure S2), and up to 0.2 g of digesta was weighed and frozen at −70 °C. DNA for microbial analyses was extracted from 200 mg of digesta samples, using magnetic beads from the MagMAX™ Total Nucleic Acid Isolation kit, using an adapted protocol on the MagMAX™ Express 96 instrument (Thermo Fisher Scientific, Waltham, MA, USA). The resulting eluted DNA was then further purified using the OneStep-96 PCR Inhibitor Removal kit (Zymo Research, Irvine, CA, USA). The DNA was then quantified on the Qubit™ 3.0 fluorometer using the Qubit™ HS kit (Thermo Fisher Scientific). Then, 1 ng of total extracted DNA was run on a QuantStudio™ 5 Real-Time PCR System using Fast Sybr mastermix (Thermo Fisher Scientific). The primers used to detect Bl-04 were: 100 nM Bl04_forward CTTCCCAGAAGGCCGGGT, 100 nM Bl04_reverse CGAGGCCACGGTGCTCATATAGA [22]. The reaction volume was 15 µL and the annealing temperature used was 60 °C, with melting curve. The assay also detects other *B. lactis* strains and melting curve analysis is required.

### 2.5. Transcriptomics

For transcriptomics, up to 100 mg of individual tissue samples were collected in TRIzol (Zymo Research) (Figure S2), homogenized by grinding beads, and RNA was extracted from the liquid supernatant using DirectZol RNA purification kits (Zymo Research) following the manufacturer’s instructions. A targeted RNA-seq library was prepared from 100 µg of RNA according to the kit instructions with TempO-Seq (BioSpyder, Carlsbad, CA, USA) mouse whole transcriptome kit for each sample. After TempO-Seq library preparation, the samples were purified, quantitated, pooled, and stored frozen prior to sequencing. The pooled libraries were sequenced using the Illumina NovaSeq platform (1 × 50 nt). The target read depth per sample was 5 million reads with the minimum reads per sample at 1 million. The reads were quality-filtered, trimmed, demultiplexed, and aligned to the BioSpyder Mouse Whole Transcriptome v. 2.0 annotated reference using Salmon (v. 1.1.0) [23]. Sample quality metrics for RNA quality and alignment to the reference can be found in Table S1.

Differential expression analysis was conducted using DESeq2 (v. 1.26.1) [24] in R (v. 3.6.2). Genes were considered differentially expressed if the adjusted *p*-value was <0.05; all differentially expressed genes (DEG) can be found in Table S2. Principal component analysis (PCA) was performed to determine the overall relatedness of the samples under the various experimental conditions, and plots were created using ggplot2 (v. 3.3.3) [25]. Pathway analysis was performed using ROntoTools (v. 2.14) [26] with a Kyoto Encyclopedia of Genes and Genomes (KEGG) pathway [27,28,29] considered significantly changed with a false discovery rate (FDR) value of <0.05.

### 2.6. Microbiota Sequencing and Analysis

To characterize the microbiota populations present in the mouse intestine, digesta was collected from the duodenum, jejunum, ileum, and caecum at Baseline (day −21; *n* = 8 per day), Week 1 (days −20, −18, −14; *n* = 8 per day), Week 3 (days −7, 0; *n* = 8 per day) pre-infection, and Week 4 post-infection (days 3, 5 and 7; *n* = 16 per day) (Figures S1 and S2). Combining timepoints according to the pre-/post-supplementation and pre-/post-infection periods (weeks) provided more appropriate sample sizes for the use of non-parametric statistical tests for the microbiota analyses. The V4 variable region of the 16S rRNA gene was amplified from the bacterial DNA using PCR barcoded primers 515F (5′-GTGCCAGCMGCCGCGGTAA) and 806R (5′-GGACTACHVGGGTWTCTAAT) under the following conditions: denaturation at 95 °C for 3 min followed by 30 cycles of denaturation at 95 °C for 45 s, annealing at 55 °C for 60 s and extension at 72 °C for 90 s, and a final extension at 72 °C for 10 min [30]. The PCR products were purified, normalized by DNA concentration, and pooled for sequencing at the W.M. Keck Center at University of Illinois–Urbana. The library was sequenced using two replicate MiSeq V2 (2 × 250 bp) runs. The sequencing data were analyzed using the QIIME2 pipeline (v. 2019.10; [31]). Reads were demultiplexed using ‘qiime demux’, and ‘qiime dada2’ was used to error-correct and dereplicate the Illumina reads using the ‘consensus’ method [32]. The reverse reads were trimmed at 180 bp and overlapping sequences were paired. Taxonomy was assigned to the amplicon sequence variants (ASVs) using ‘q2-feature-classifier’ (classify-sklearn) trained on the V4 region of sequences contained in the RDP Classifier training set No. 18 (v. 2.13; July 2020 release) [33,34]. Taxa compositions are reported as relative abundance (% of total sequences). Samples that did not contain at least 10,000 sequences were removed from the study analyses. For all diversity metric calculations, the samples were normalized by rarefaction to 10,000 sequences per sample. Alpha (within sample) diversity was calculated according to Faith’s Phylogenetic Diversity (PD) metric [35], and beta diversity (between sample pairwise dissimilarity) was calculated using the weighted UniFrac metric [36]. Study groups were compared by permutational multivariate ANOVA (PERMANOVA) using the R v. 3.6.1 ‘vegan’ package adonis test [37]. Differentially abundant taxa were tested between groups using the R package ‘ANCOM2’ on ASVs greater than 0.1% total abundance for the main effect of ‘Group’ and including ‘Cage’ as a random effect [38]. The *p*-values were adjusted according to the Benjamini–Hochberg method and a significance cutoff of *p* < 0.05 after correction was used [39].

### 2.7. Statistics

The statistical analyses for the viral load and mouse health metrics were carried out at 4Pharma Ltd. (Turku, Finland) using SAS^®^ System for Windows, version 9.4 (SAS Institute Inc., Cary, NC, USA). The viral load measurements were first log10-transformed for statistical analysis. The transformed values were analyzed with a linear mixed-effects model where treatment, study day, location (left vs. right lung), and the interaction between treatment and study day were included as fixed effects and mouse as the random effect in the model. The differences (together with 95% CIs) between the treatments within study day were estimated from the model using contrasts.

The difference between the treatment groups in the percentage change from baseline in body weight was analyzed using a linear mixed-effects model where treatment, study day, and the interaction between treatment and study day were included as fixed effects and mouse as a random effect in the model. An estimate +95% CI for the overall difference between the treatments was calculated from the model.

The incidence of rough coat, abnormal breathing, and hunched posture were all analyzed, with mixed-effects logistic regression models between study days 3 and 7. The model included treatment, study day, and the interaction of treatment and study day as fixed effects. Mouse was included as the random term. In the logistic regression analyses, ORs and their 95% CIs were used to quantify the results within the study day. *p*-values < 0.05 were considered statistically significant and were not adjusted for multiple testing.

## 3. Results

### 3.1. Bl-04 Decreases Viral Load and Modestly Improves Health Scores in H1N1-Infected Mice

To evaluate the effect of the probiotic on H1N1 IAV infection, Bl-04 at 2 × 10^9^ CFU per day or placebo was administered to mice 21 days prior to (T-21) and 13 days post (T13) infection (T0), and tissues and digesta were collected for further analyses (Figures S1 and S2).

**Figure 1:**
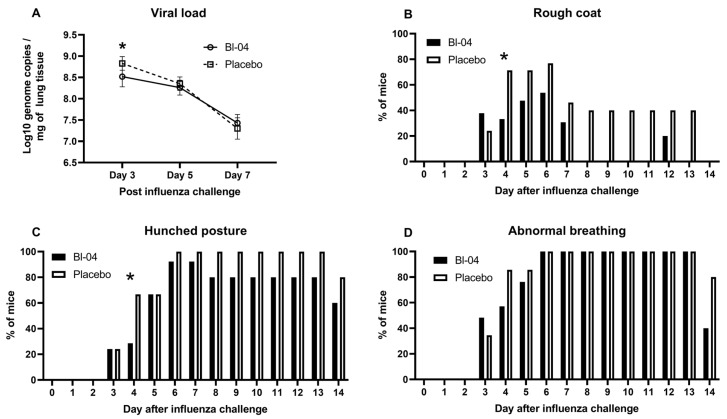
Viral load and health scores in IAV-infected mice. (**A**) Mean +/− SD IAV H1N1 log_10_ genome copies per mg of mouse lung tissue measured by RT-qPCR at days 3, 5, and 7 post-infection in Bl-04 (*n* = 8) and placebo (*n* = 8) groups. * Estimated difference for linear mixed-effects model for log10-viral load significant at day 3 (*p* = 0.0004). (**B**) Percentage of mice with rough coat at different study days in Bl-04 and placebo groups. * Odds ratio for the treatment difference between groups significant at day 4 (*p* = 0.046). (**C**) Percentage of mice with hunched posture at different study days in Bl-04 and placebo groups. * Odds ratio for the treatment difference between groups significant at day 4 (*p* = 0.039). (**D**) Percentage of mice with abnormal breathing at different study days. For health scores, the number of animals per group was 29 for days 0–3, 21 for days 4–5, 13 for days 6–8, and 5 for days 9–14.

First, we investigated the effect of Bl-04 treatment on the H1N1 viral load. Left lobes of the lungs were collected at T3, T5, and T7 post-infection and analyzed by H1N1-specific RT-qPCR. H1N1 viral loads in the lungs of both Bl-04 and placebo groups were highest at T3 (Figure 1A). The average viral load at T3 of the Bl-04-treated mice was 3.72 × 10^8^ genome copies/mg of tissue compared to 7.17 × 10^8^ genome copies/mg of tissue in the lungs of the placebo group. The viral load at T3 was significantly lower (numerically 3.45 × 10^8^ copies/mg or 48%) in the Bl-04 group than in the placebo group (estimated difference from the linear mixed-effects model for log10-viral load −0.32; 95% confidence interval (CI) −0.48, −0.16; *p* = 0.0004). Viral loads in the lungs were, on average, lower at T5 and further at T7 in both groups. No significant differences in the viral loads were found between the two groups at T5 or T7.

To further evaluate the effects of Bl-04, the mice in each group were evaluated for rough coat (Figure 1B), hunched posture (Figure 1C), or abnormal breathing (Figure 1D) indicated by increased rates of respiration. The number of mice evaluated decreased during the study progression due to mice being sacrificed for sampling (T1–T3 = 58, T4–T5 = 42, T6–T7 = 26; T8–T14 = 10; Figure S1). The earliest of these symptoms developed at T3 post-infection (Figure 1B–D). Abnormal breathing and hunched posture persisted in some mice through T14, while rough coat was resolved in all remaining mice by T14. Rough coat was observed less frequently in the Bl-04 group animals between T4 and T14 post-infection and was not observed in any of the Bl-04-treated animals on T8, T9, T10, T11, T13, and T14 post-infection (Figure 1B). At T4, 7 of 21 mice in the Bl-04 group and 15 of 21 mice in the placebo group had rough coat and the difference between the groups reached statistical significance (odds ratio (OR) 0.12; 95% CI 0.02, 0.97; *p* = 0.046). Similarly, a smaller proportion of mice in the Bl-04 group had hunched posture at T4 compared to the placebo group (OR 0.14; 95% CI 0.02, 0.90; *p* = 0.039) (Figure 1C). The number of Bl-04 group animals with hunched posture was equal to or less than that observed in the placebo group animals for the entirety of the study. Abnormal breathing was observed in 100% of the mice from both groups between T6 and T13, but resolved in three mice in the Bl-04 group and one mouse in the placebo group on T14 (Figure 1D). In addition to the abnormal breathing described above, one mouse in the Bl-04 group developed dyspnea on T3 post-infection, after which it was resolved. In contrast, three mice in the placebo group developed dyspnea. Labored and accelerated breathing were the most severe respiratory symptoms observed during the study.

The average body weight of both groups dropped sharply between T2 and T4 post-infection (Figure S3). Mice in the Bl-04 group and the placebo group reached peak body weight loss on T6 post-infection at 11.1% and 11.9%, respectively. The average body weight loss improved each day between T6 and T13 post-infection, at which point the average body weight of both groups reached baseline (T-21) levels. In the analysis of the percentage change from the baseline of the body weight, no overall difference was observed between groups (estimated overall difference 0.47; 95% CI −0.97, 1.90; *p* = 0.522).

### 3.2. Bl-04 Does Not Have a Broad Influence on Tissue Transcriptomes

**Figure 2:**
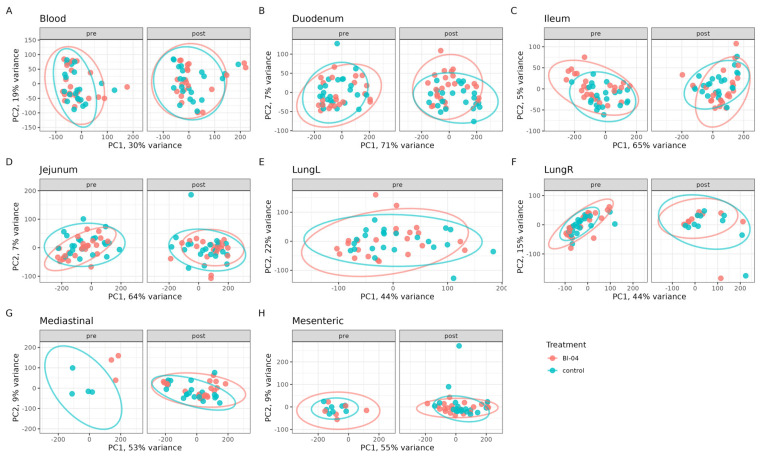
Global transcriptomics analyses of Bl-04 and placebo groups in all tissues tested over the course of the study. The data points of the same analysis are divided into pre- and post-infection timepoint panels. Principal component analysis (PCA) of transcriptomes of all samples tested in Bl-04 (red) and placebo control (blue) group. Tissues are separated into individual panels (**A**–**H**). Each point is an individual sample transcriptome containing all genes expressed. Ellipses represent a 95% confidence level for a multivariate t-distribution.

To determine the effect of Bl-04 on the mouse transcriptome pre- and post-H1N1 infection, RNA was extracted and sequenced from the duodenum, jejunum, ileum, lymph nodes (mesenteric and mediastinal), blood, and lungs at study days T-21, T-20, T-18, T-14, T-7, T0, T3, T5, and T7 (Figure S1). Small intestinal tissues were included in the analyses because the orally fed probiotic was expected to influence the host via the epithelial or immune cells in the small intestine where the probiotic is more likely to come into direct contact with host cells compared to the large intestine [40]. Some samples are missing from the data analysis, as the small size of the pre-infection lymph nodes made their collection challenging and the left lung lobes were used for viral RT-qPCR. We conducted a principal component analysis (PCA) on all DEGs that were determined. Overall, the smallest amount of variation explained by the first two principal components was observed in the blood samples, and the largest amount of variation was captured in the duodenum samples (Figure 2). There was no clear separation of the clusters by the treatment groups, showing that Bl-04 did not have a broad influence on the transcriptomes (Figure 2).

We looked further into the number of DEGs at different timepoints and tissues between the Bl-04 and placebo groups (Figure S4). The DEG analyses showed, unexpectedly, a high number of downregulated DEGs in the ileum, jejunum, and left lung, but not the other samples between the Bl-04 and placebo groups at baseline timepoint T-21 (Figure S4). In addition, at T0, the duodenum, lungs, and mediastinal lymph nodes showed higher amounts of DEGs between the treatment groups than at the other pre-infection timepoints. Post-infection, a difference in the DEGs between the Bl-04 and placebo groups was observed in all timepoints, most prominently at T5, when the blood, ileum, jejunum, and right lung samples had higher numbers of DEGs in the Bl-04 group compared to the placebo group (Figure S4). The large variation in DEGs, especially at T-21 (when no treatment was given) and T0 (absence of treatment effect in the previous timepoints T-20, T-18, T-14, T-7), indicate in vivo experimental variation or potential technical errors during the sample processing. Thus, for T3, T5, and T7, it is difficult to conclude how much the results (see below) were influenced by technical variation and how much by the treatments.

### 3.3. Effect of Bl-04 on Influenza A and Immune Pathways

Although we could not detect effects on the whole transcriptome, as suggested by the DEG analysis and PCA, Bl-04 could potentially modulate the genes involved in the host response to IAV infection. To understand more broadly which signaling pathways were modulated by Bl-04 before and during H1N1 infection, we conducted a pathway analysis, which calculates the probability of a KEGG pathway being “perturbed” (that may mean the inhibition or activation of the pathway or its parts) using a threshold of log2 fold changes and *p*-values for all genes in our dataset.

**Figure 3:**
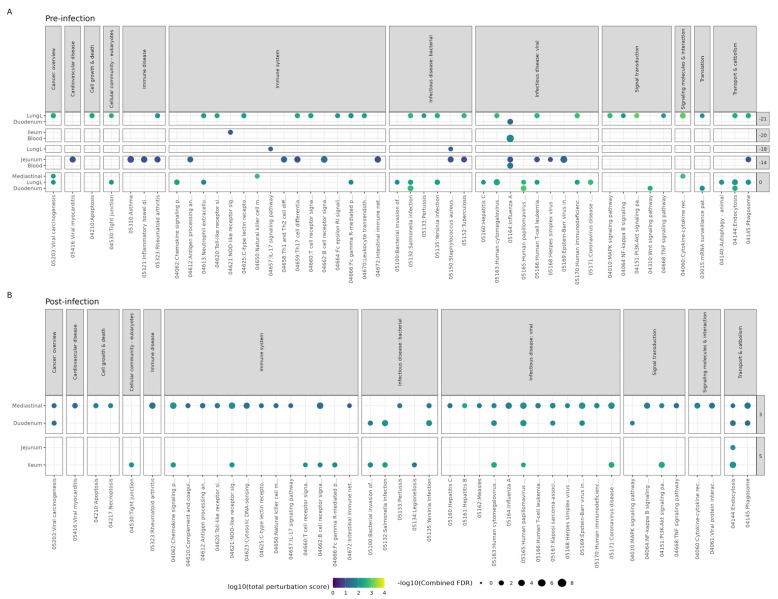
Pathway analysis results of Bl-04 treatment group compared to the placebo group. ROntoTools pathway analysis using differential expression comparing Bl-04 to placebo group within each timepoint and tissue at (**A**) pre-infection and (**B**) post-infection. All points shown are significant (combined FDR < 0.05), with the color denoting the total level of pathway perturbation and the size denoting the significance. Pathways from selected categories are shown. All pathways can be found in Table S2. Any timepoint or tissue not shown is due to no significant pathway perturbation.

Prior to infection, 163 unique pathways were perturbed (with combined FDR < 0.05) in the blood, lungs, duodenum, ileum, jejunum, and mediastinal lymph nodes (Table S3). The timepoint before any supplementation (T-21) had the most perturbed pathways (112), followed by T0, T-14, T-20, and T-18 with 107, 35, 6, and 5 perturbed pathways, respectively. The Influenza A pathway (Figure 3A) was perturbed at three timepoints before infection, in duodenum at T-21, in blood at T-20, and in jejunum and blood at T-14.

Post-infection, the Bl-04 group mice had several viral infectious disease pathways affected in the duodenum at T3 and the ileum at T5 (Figure 3B). Only the intestinal tissue and mediastinal lymph node tissue differed between the Bl-04 and placebo groups at T3 and T5, and there were no differences between the groups at T7. The Influenza A pathway was shown to be perturbed by Bl-04 in mediastinal lymph nodes at T3 compared to placebo (combined FDR = 0.00028) (Figure 3B). Hypothetically, the modulation of immune-specific pathways and the upregulation of antiviral genes by Bl-04 administration post-infection could increase the mouse’s ability to fight viral infection. This activation of many innate resistance pathways could lead to the reduction in the viral titer observed in the Bl-04 group compared to the placebo group.

**Figure 4:**
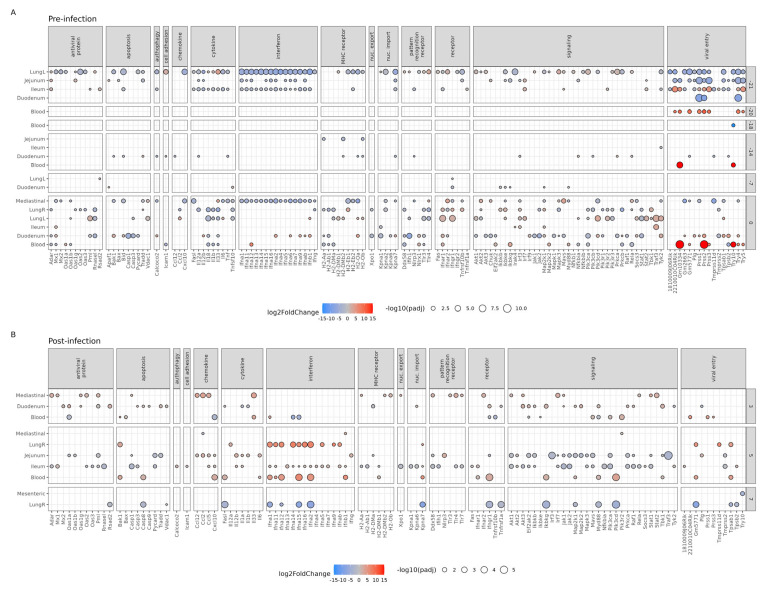
Influenza A pathway gene expression prior to and during H1N1 infection in mouse tissues. Balloon plot of Influenza A (mmu05164) KEGG pathway genes found differentially expressed in the Bl-04 group compared to placebo group at (**A**) pre-infection and (**B**) post-infection timepoints. All genes shown have significant expression (padj < 0.05). Size of the balloon denotes significance (−log10-adjusted *p*-value). Color denotes expression level (log2 fold change) with blue having lower expression and red having increased expression relative to the placebo group. LungL: left lung, LungR: right lung.

To further study the impact of the Bl-04 on the host genes involved in the IAV life cycle and host innate immune response pre- and post-infection, we analyzed the DEGs in the KEGG pathway for Influenza A (mmu05164) between the Bl-04 and the placebo group at each timepoint and tissue (Figure 4). Pre-infection (Figure 4A), the mice sacrificed (*n* = 4 per group) at different timepoints differed in the magnitude of Influenza A pathway gene expression and no consistent gene expression profile per tissue could be detected. The perturbations of the Influenza A pathway at T-21, T-20, and T-14 (Figure 3A) seem to be driven by DEGs in the MHC receptor and viral entry categories (Figure 4A) and, thus, do not suggest immune activation by Bl-04 or IAV. At T-21, before any treatment, lung, duodenum, jejunum, and ileum sample DEGs showed the downregulation (overall 90% (158/176) downregulated) of many cytokine, interferon (IFN), signaling, and viral entry genes between the groups that likely reflects technical variation, as discussed above. At days T-20 and T-18, the only difference between the Bl-04 and placebo groups was seen in the blood in the expression of viral-entry-related genes. At T-14 and T-7, the duodenum samples showed a sporadic regulation of genes in several gene categories. At T0, most tissues, such as mediastinal lymph nodes, both lungs, ileum, duodenum, and blood, and many gene categories in the Influenza A pathway were regulated in the Bl-04 group compared to the placebo group. Of all DEGs at T0, 38% (96/255) showed upregulation, and of those reflecting antiviral immunostimulation (antiviral protein, chemokine, cytokine, and interferon categories), 24% (20/83) were upregulated. Of the mediastinal DEGs, 16% were upregulated, whereas lungL (62%), lungR (23%), ileum (83%), duodenum (48%), and blood (47%) samples had more upregulated DEGs. The effect of the probiotic at T0 cannot be excluded; however, the absence of effects at T-20 through T-7 suggests that the result is driven by technical variation. Overall, and excluding the T0 DEGs, the probiotic does not seem to regulate the Influenza A pathway pre-infection.

Post-infection at T3, more upregulated DEGs were detected across all (57% (43/75)) and antiviral immunostimulation (70% (16/23)) categories in comparison to T0. The increase in DEG upregulation was reflected in the gene categories shared between T0 and T3, where at T3, the mediastinal (100%), duodenal (80%) and blood (59%) DEGs were upregulated by the probiotic. A similar trend continued at T5, where 57% (81/141) of the total DEGs and 84% (47/56) of the antiviral immunostimulation DEGs were upregulated. At T7, 0% (0/19) of the DEGs were upregulated, suggesting a loss of effect, in line with no difference in viral load or symptom scores. Thus, at T3 and T5, the Bl-04 group DEGs showed a general upregulation in contrast to the pre-infection and T7 timepoints. Post-infection, more mice were sacrificed per group (*n* = 8) than pre-infection (*n* = 4), thus increasing the reliability of the results from these timepoints. Further, the induction of innate response genes is in line with the bacterial stimulation of immunity, in contrast to T-21 and T0, where these DEGs were generally downregulated. The results suggest an effect of Bl-04; however, due to the technical variation, the interpretation is not conclusive.

Of the specific genes of interest, at T3, the mediastinal tissues from animals treated with Bl-04 showed the upregulation of several antiviral protein genes compared to placebo such as *Adar*, *Mx1*, *Oas1g*, *Oas2*, and *Pml*, MCH-receptor genes, pattern recognition receptor (PRR) genes *Ddx58*, *Tlr3*, *Tlr4*, and *Tlr7*, and signaling genes such as *Akt1*, *Akt3*, *Irf7*, *Jak1*, *Rela*, *Stat1*, and *Stat2* (Figure 4B). Also, the duodenum showed the upregulation of genes in the antiviral protein, apoptosis, chemokine, and signaling categories. The largest difference was shown at T5, when several IFN-α genes and cytokine genes in blood and lung tissues were upregulated in H1N1-infected Bl-04 group mice compared to the placebo group. Intestinal samples showed the downregulation of antiviral and signaling genes and the upregulation of chemokine, cytokine, and IFN genes. At T7, IFN and signaling genes were downregulated in the Bl-04 group’s lung tissue compared to that of the placebo group mice.

Considering the technical variation (Figure S4) in the DEG numbers across the timepoints, especially at T-21 and T0, it is difficult to draw conclusions about the probiotic effect compared to pre-infection. The increase in upregulated DEGs related to IAV defense at T3 and T5, in contrast to T-21, T0, and T7, that coincide with a decrease in viral load and symptom scores, leads to a hypothesis of immunostimulation by Bl-04 that should be tested in further experiments to obtain conclusive evidence.

### 3.4. Bl-04 Does Not Cause Major Shifts in the Murine Intestinal Microbiota Composition

**Figure 5:**
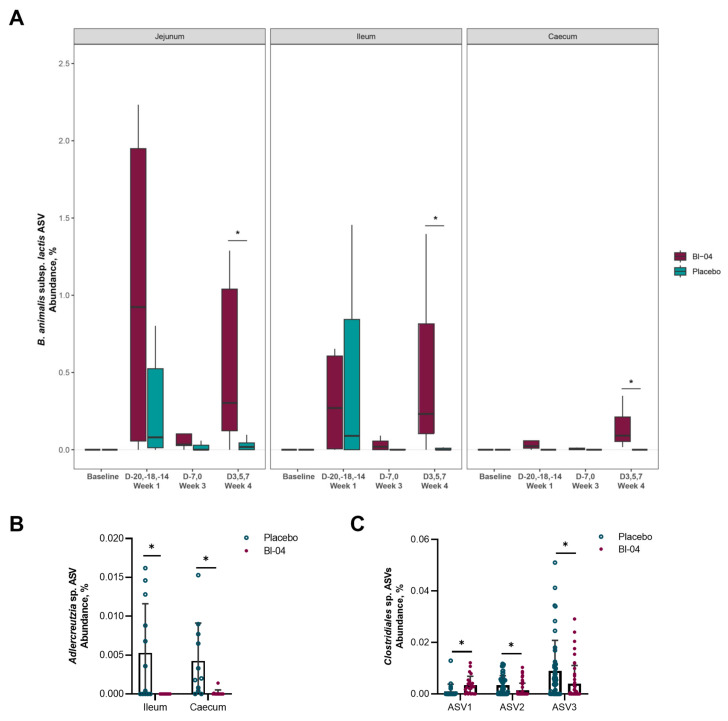
Differentially abundant amplicon sequence variants (ASVs) in the intestinal microbiota. The ASV corresponding to the subspecies *B. animalis* subsp. *lactis*, including Bl-04, was increased overall in the jejunum, ileum, and caecum (all weeks combined for each intestinal section; FDR < 0.05) and at Week 4 for each intestinal section (* FDR < 0.05) compared to placebo (**A**). An ASV corresponding to Adlercreutzia sp. was increased at Week 1 in the ileum and caecum of placebo mice compared to Bl-04 (* FDR < 0.05) (**B**) and three ASVs corresponding to *Clostridiales* sp. (novel species) were differentially abundant in the caecum at Week 4 (* FDR < 0.05) (**C**). Baseline (day −21; *n* = 8 per day), Week 1 (days −20, −18, −14; *n* = 8 per day), Week 3 (days −7, 0; *n* = 8 per day) pre-infection, and Week 4 post-infection (days 3, 5, and 7; *n* = 16 per day).

To characterize the microbiota populations present in the mouse intestine, digesta was collected from the duodenum, jejunum, ileum, and caecum (Figure S2). The samples were grouped according to baseline prior to probiotic supplementation (T-21; *n* = 8 per day), Week 1 post-probiotic supplementation (T-20, T-18, T-14; *n* = 8 per day), Week 3 (T-7, T0; *n* = 8 per day) prior to infection, and Week 4 post-infection (T3, T5 and T7; *n* = 16 per day) (Figure S1). We found it important to include samples from different sites of the intestine as the gut microbiota has been traditionally analyzed from the feces, even though the microbiota populations differ throughout the gut [41]. Samples that did not produce sufficient sequencing data for analysis were removed from the study. These samples were disproportionately taken from the small intestine, and not all samples produced PCR amplicons due to low microbial biomass. The alpha (within-sample) species diversity did not differ significantly between the placebo and Bl-04 groups in any of the intestinal sections (*p* > 0.05; Mann–Whitney U test) (Table S4). The samples did not cluster significantly according to study group in the small intestinal sections by the beta diversity weighted UniFrac metric (*p* > 0.05; PERMANOVA); however, there was small, but significant, clustering by study group in samples from the caecum (R^2^ = 0.02, *p* = 0.001; PERMANOVA) (Table S5). The largest contributing factor to sample clustering for all intestinal sections was timepoint (R^2^ > 0.23, *p* = 0.001; PERMANOVA), and the interactions between timepoint and study group were not significant (*p* > 0.05) (Table S5). The relative abundance of the amplicon sequence variant (ASV) corresponding to the subspecies *B. animalis* subsp. *lactis* was significantly higher in the Bl-04 group compared to placebo in the jejunum, ileum, and caecum (overall and at Week 4, FDR < 0.05; ANCOM) (Figure 5A). The species diversity and CFU/mL of microbes in the jejunum and ileum (10^4^–10^7^) are known to be lower than in the caecum (10^9^); thus, the relative abundance of *B. animalis* subsp. *lactis* in the different locations cannot be directly compared to each other. Notably, it does not appear that there was a consistent colonization of Bl-04 until the post-infection period. There was an increased abundance of *Adlercreutzia* sp. at Week 1 prior to infection in the ileum of the placebo compared to the Bl-04 group, and it was similarly increased during this time in the caecum (FDR < 0.05; ANCOM) (Figure 5B). There was no other substantial difference in taxa abundances between the placebo and Bl-04 groups in the small intestine. In the caecum, the Bl-04 group had an increased abundance of *Clostridiales* sp. (ASV1) at Week 4 post-infection compared to placebo and an overall decreased abundance of two *Clostridiales* sp. (ASV2 and ASV3) (FDR < 0.05; ANCOM) (Figure 5C).

### 3.5. H1N1 Infection Associated with Higher Prevalence of Bl-04 in the Intestine

The presence of the Bl-04 strain in the mouse intestine was assessed by strain-specific qPCR from digesta collected from the duodenum, jejunum, ileum, and caecum at each timepoint (Figure S5). In the Bl-04 group, Bl-04 was not detected in any of the samples at days T-21, T-20, T-14, but at T-18 (Week 1), most of the samples from two mice gave a positive signal, one mouse showed a signal in the jejunum, and one mouse was negative. At T-7 (Week 3), one mouse in the Bl-04 group had a positive ileum sample, and at T0 (Week 3), one mouse had a positive caecum sample. The placebo group did not show a signal for Bl-04 at any timepoint, except at T-18 (Week 1), when, inexplicably, one mouse had three positive samples and another mouse had one. At post-infection T3, T5, and T7 (Week 4), most of the Bl-04 group caecum samples and sporadic samples in other intestinal sites were positive for Bl-04, indicating that the influenza infection changes the gut environment or microbiota favoring Bl-04 colonization (Figure S5). The Bl-04 qPCR results were well aligned with the 16S rRNA ASV result (Figure 5A).

## 4. Discussion

The meta-analyses of human clinical studies suggest that probiotics could potentially reduce the incidence of upper respiratory tract infections [9,11]; however, the results of the clinical studies seem to depend on the strain and are challenging to reproduce. A more in-depth understanding of the strain-specific mechanisms of action of probiotics on the immune system and microbiota is needed. In this study, we treated mice with Bl-04 or placebo and challenged them subsequently with H1N1 IAV. The results show a decrease in the lung viral load by 48%, and a small improvement in health scores by Bl-04.

Our study results showed that the treatment of mice with Bl-04 significantly lowered the H1N1 load in the lungs at T3 (Figure 1A) and improved the health of mice at T4, as measured by rough coat and hunched posture (Figure 1C,D). These data add to the evidence from previous pre-clinical and clinical studies showing that the supplementation of probiotics strain-dependently reduces IAV load in the lungs of mice and improves health scores [17]. In previous studies, the intranasal administration of two *Bifidobacterium longum* strains [42] or the oral administration of *B. longum* MM-2 [43] or *Lactobacillus gasseri* LG2055 [44] to mice prior to infection was shown to reduce viral load and cytokine levels after exposure to the mouse-adapted influenza virus strain PR8. A recent study showed a 61% decrease in lung viral load at day 3 post-infection and no effect on day 7 post-infection by *Bacteroides dorei* [45]. Our study is the first to show that a live probiotic can reduce the viral load in mice infected with a human pandemic A/California/07/2009 virus strain. In a mouse study with a lethal mouse-adapted A/California/04/2009 virus strain, the authors showed a better survival in mice orally gavaged with heat-killed *Lactobacillus pentosus* b420 [46]. However, they did not detect a difference in the lung viral load or lung gene expression between the groups, raising the question of whether an intact probiotic is more efficacious. In this study, we supplemented the mice by gavage, but also by oral exposure using cereals soaked with Bl-04; thus, the effect on H1N1 could be due to probiotic influence on the gut–lung axis or via more direct effects in the upper respiratory tract. In the previously published studies, probiotic application via the nasal route [47,48,49,50] or gavage [43,44,51,52,53] has been shown to be effective in IAV mouse models.

For Bl-04, the reduced incidence of upper respiratory tract illness episodes in adults has been documented [13,54], which supports the findings of this study. On the other hand, in a human rhinovirus challenge study, the reduced incidence of rhinovirus infection or decreased viral load was not observed when Bl-04 was supplemented prophylactically [16]. However, in a previous rhinovirus challenge study, Bl-04 supplementation decreased rhinovirus load and detection prevalence in nasal washes of the subjects [15]. Thus, it seems that the effects of Bl-04 in clinical studies are difficult to reproduce, potentially due to the probiotic causing the mild stimulation of the immune system. In the current study, the effects on the influenza viral titer in the lungs and health scores (Figure 1) were small and in line with the clinical data [13,15,16].

We aimed to discover immune pathways and genes associated with Bl-04 treatment pre-infection and post-infection. Pre-infection, we observed a broad downregulation of genes at T-21 when no treatment was applied and at T0 without effect in other treatment timepoints, suggesting technical variation in the in vivo study or subsequent sample processing. With higher number of samples post-infection, we observed the upregulation of genes related to IAV defense and bacterial stimulation at T3 and T5, suggesting immunostimulation by Bl-04. However, we cannot conclude this, as some of the effect may be driven by technical variation. Thus, the specific effects of Bl-04 on anti-viral immunity are inconclusive, but can be used for hypothesis generation in further studies. Given the technical issues, the results of the pathway and DEG analyses at T3 and T5 lead to the hypothesis that Bl-04 may influence the immune response to H1N1 in an alignment with decreased viral load at T3 (Figure 1A) and health scores at T4 (Figure 1B,C). The potentially modulated pathways and DEGs at T3 and T5 were found in the small intestine, one of the intestinal sites for probiotic activity, and in the mediastinal lymph nodes, where the Influenza A pathway was significantly perturbed at T3 (Figure 3). We further looked at genes that could explain the effects on the viral load. Overall, and given the technical variation, at T3 and T5, there seemed to be a broad upregulation of DEGs related to innate immunity and anti-viral defense by Bl-04. After IAV challenge, type I and II IFN genes were upregulated at T5 in the blood, ileum, and lung samples by Bl-04 (Figure 4B). IFNs are essential in the host response to viruses [55], so their increased activation could explain the beneficial effects of Bl-04 against the virus. IFNs are induced via the recognition of pathogen-associated molecular patterns (PAMPs) by PRRs such as Toll-like receptors and RIG-I-like receptors. IFN production, in response to influenza, is predominantly mediated by RIG-I [56,57]. At T3, the probiotic treatment induced an upregulation of *Ddx58* (RIG-I), as well as the PRRs *Tlr3*, *Tlr4*, and *Tlr7*, which bind to double-stranded RNA (dsRNA), lipopolysaccharides (LPS) of various bacteria, and single-stranded RNA (ssRNA), respectively (Figure 4B). Commensal bacteria have various RNA transcripts, which may mimic PAMPs that are detected by TLRs to drive IFN activation [58]. Furthermore, studies have demonstrated that bifidobacteria can inhibit LPS-induced NF-κB activation and inflammation [59,60]. Interestingly, type III IFN genes were not similarly upregulated, despite reports that they are triggered by the same viral components as type I IFNs [61]. In summary, the increased expression of IFN genes by Bl-04 treatment after early PAMP detection by multiple receptors may be a key factor involved in the reduction of viral load and adverse health scores, but given the technical variation, these hypotheses must be confirmed in subsequent studies.

The Bl-04 treatment did not broadly affect the murine intestinal microbiota composition, as analyzed by 16S rRNA sequencing (Figure 5). This result is in agreement with clinical study findings in healthy humans where Bl-04 did not substantially change the fecal microbiota composition after 4 weeks of supplementation [15,16]. We observed a small difference in the microbiota composition between the study groups, where the placebo group had a greater abundance of *Adlercreutzia* sp. in both the ileum and caecum at Week 1 (Figure 5B); however, the overall abundance of this species in the microbiota was very low and not likely to be an impactful finding. When we specifically targeted the detection of Bl-04, the probiotic strain was found in the small intestine and caecum of most of the mice in the Bl-04 group at Week 1 by sequencing (Figure 5), and at T-18 by qPCR (Figure S5). Interestingly, at Week 3, Bl-04 was still mostly undetected, which could indicate colonization resistance for Bl-04 in mice by the endogenous microbiota. Bl-04 was consistently detected and most abundant in the caecum of the probiotic-supplemented mice during the post-infection period. The quantitative strain-specific Bl-04 qPCR results were well aligned with the relative abundance of the ASV corresponding to the subspecies-level detection of *B. animalis* subsp. *lactis*, which also indicates that this taxon was not prevalent in the endogenous microbiota. In comparison, transcriptomics analysis of the Bl-04 group mice found most DEGs impacted in jejunum and ileum at Week 1 (T-20, T-18, T-14), but not at Week 3 (T-7) (Figure 4A). These findings suggest, but are not conclusive, that there was an innate immune response to the probiotic at Week 1 that was potentially followed by adaptive immune responses, thereby generating IgA and re-establishing homeostasis, and explaining the fewer DEGs and lower detection of Bl-04 at Week 3. In the fecal samples collected from a human clinical study, Bl-04 was detected from 83% [16] and 69% [15] of the subjects taking the investigational product, in line with the differences in the detection in mice.

In the microbiome analysis, the largest contributing factor to sample clustering for all intestinal sections was timepoint, and the interactions between the timepoint and study group were not significant, indicating that there may be a time-dependent shift in the microbiota that is not attributed to Bl-04 supplementation. A plausible reason is that the microbiota is influenced over time by the course of the influenza infection, as shown by others [62,63]. It has been shown that the microbiota of H7N9-infected mice was different between mice that died due to the infection and mice that survived [64], and in particular, *B. animalis* and *B. pseudolongum* levels were elevated in the feces of the mice that survived. The authors also showed that treating the mice with *B. animalis* increased the survival of the mice infected with the H7N9 influenza virus. In the microbiota analyses, we found a higher prevalence of Bl-04 during the infection stage in the digesta samples using sequencing (Figure 5A) and qPCR (Figure S5). The reason for the colonization during infection, but not before, is not evident, but most likely is beneficial for the host and may have a similar mechanism of action that results in the increase in the endogenous bifidobacteria during influenza infection [64]. We additionally observed differential abundances for three different ASVs classified as *Clostridiales* sp. in the caecum at Week 4 post-infection between the Bl-04 and placebo group (Figure 5C). None of these ASV sequences had matches greater than 98% similarity to the reference organisms in public taxonomic databases, which indicates that they are likely novel species [65]. It is interesting to note that the differential responses in these ASVs occurred between the groups in the post-infection period, as it also coincides with where the levels of the Bl-04 strain were detected more consistently in the mice. Due to the uncharacterized nature of these species, it unfortunately remains difficult to speculate what their function or specific role may be.

The DEG analyses suggested a regulation of immune pathways (Figure 3B) and upregulation of genes (Figure 4B) post-infection in the small intestine and lungs by Bl-04. Thus, the data associate the regulation of small intestinal and lung tissues during upper respiratory tract infection and probiotic prevalence in the gut, but whether that is of significance to H1N1 infection or caused by H1N1 infection remains to be determined. Of note, similar results were observed in a SARS-CoV-2 challenge study in ferrets with a probiotic blend containing Bl-04, where the authors showed the regulation of SARS-CoV-2 receptor ACE2 and immune response genes in the duodenum and lungs during the infection [66].

This study has several limitations. The viral load was analyzed by RT-qPCR; however, a TCID_50_ or a plaque-forming assay could have been conducted to confirm the live virus amounts in the lungs of the mice. One option would have been to analyze the viral protein, e.g., nucleoprotein levels in the lungs, using histology or fluorescence microscopy. The transcriptomics results showed unexplained differences in the gene expression levels between the mice at different timepoints and a repeat experiment should have been conducted to confirm and interpret the results. For specific genes, the gene expression results should have been confirmed by RT-qPCR, and the IFN gene expression from blood could have been confirmed at protein level using ELISA. We wanted to analyze samples from the small intestine as we hypothesize that it is the site where the probiotic most likely comes into contact with immune cells and/or gut epithelial cells and can influence innate immunity. The microbiota of the colon, where the conditions are the most appropriate for bifidobacterial metabolism, could have been analyzed for comparison. Also, analyses of fecal or digesta metabolites could have revealed functional differences between the microbiota of the mice not shown by the 16S sequencing.

In summary, treatment with the probiotic Bl-04 decreased the Influenza A H1N1 viral titer and mildly improved the symptom scores associated with the infection. Based on the transcriptomics results, we cannot conclude how Bl-04 modulates the IAV infection response via the immune system. This warrants more precise methods. Moreover, the microbiota composition in the small intestine or caecum did not explain the antiviral effect of Bl-04. Further pre-clinical and clinical studies utilizing molecular methods are required to elucidate the mechanism of action of Bl-04 in viral infections.

## Data Availability

The 16S rRNA amplicon and transcriptomics datasets generated during the current study are available in the NCBI SRA repository under BioProject PRJNA949701.

## Supplementary Materials

The following supporting information can be downloaded at: https://www.mdpi.com/article/10.3390/microorganisms11102582/s1, Figure S1: Influenza challenge study set-up and sampling; Figure S2: Sectioning of mouse intestine for microbiota and transcriptomics sampling. Figure S3: Mouse body weights over the course of the study; Figure S4: Differentially expressed genes (DEGs) in different mouse tissues in Bl-04 group compared to placebo group at each timepoint; Figure S5: Heatmap showing qPCR estimates of Bl-04 genomes/nanogram of digesta from different tissues of placebo-treated (a) and Bl-04-treated (b) mice; Table S1: Table of mapping results from quantifying probes using Salmon against the BioSpyder mouse probeset. Table S2: Filtered DEG table—Excel file; Table S3: Filtered pathway table—Excel file; Table S4: Alpha diversity (Faith’s Phylogenetic Diversity (PD) metric) comparisons between placebo and Bl-04 study groups; Table S5: PERMANOVA (adonis) test describing the effect of study factors on beta diversity sample clustering from intestinal microbiota samples; Table S6: Excel data file containing microbiota abundance profiles for all samples.
