# Supplementary material for: The Effect of Bifidobacterium animalis subsp. lactis Bl-04 on Influenza A Virus Infection in Mice
Source: Microorganisms. 2023 Oct 17;11(10):2582. doi: 10.3390/microorganisms11102582 (PMC10609243; doi:10.3390/microorganisms11102582)
Supplement: Supplementary file 1 [file microorganisms-11-02582-s001.zip › microorganisms-2632067-supplementary.pdf]

microorganisms

MDPI

Supplementary Material

The Effect of *Bifidobacterium animalis* subsp. *lactis* Bl-04 on Influenza A Virus Infection in Mice

Bryan Zabel [1,†], Sanna M. Mäkelä [2,*†], Derek Nedveck [1], Ashley A. Hibberd [1], Nicolas Yeung [2], Sinikka Latvala [2], Liisa Lehtoranta [2], Jouni Junnila [3], Kevin B. Walters [4], Wesley Morovic [1], Markus J. Lehtinen [2]

[1] Health & Biosciences, International Flavors & Fragrances, 3329 Agriculture Dr., Madison, WI, 53716, USA
[2] Health & Biosciences, International Flavors & Fragrances, Sokeritehtaantie 20, FI-02460 Kantvik, Finland
[3] 4Pharma Ltd, Tykistökatu 4D, FI-20520 Turku, Finland
[4] Department of Infectious Disease Research, Southern Research Institute, 431 Aviation Way, Frederick, MD 21701, USA
* Correspondence: sanna.makela@iff.com;
† These authors contributed equally to this work.

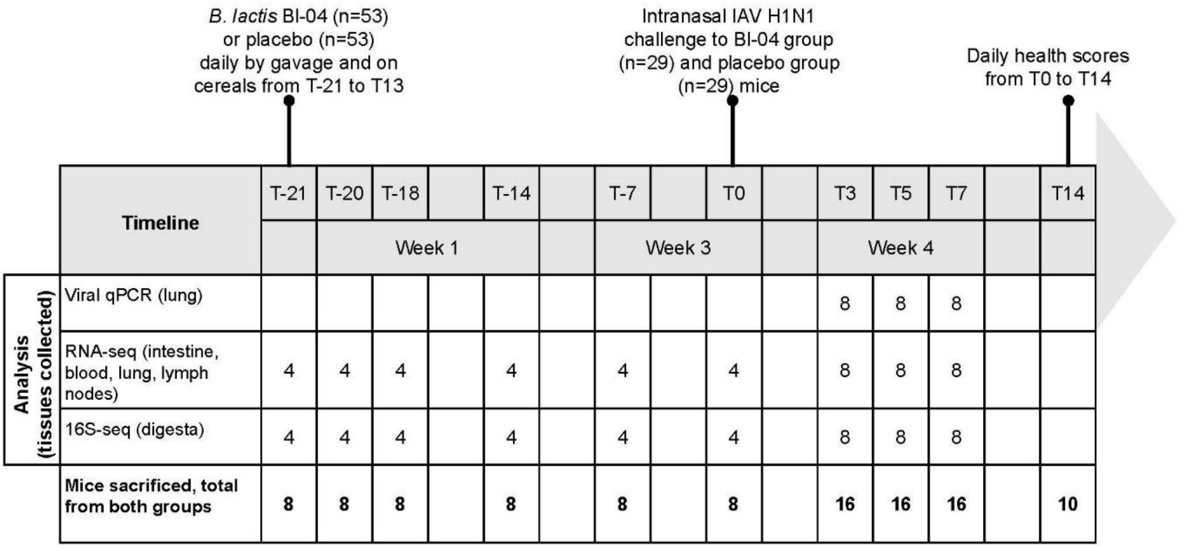

Figure S1. Influenza challenge study set-up and sampling. The table indicates the number of mice collected for specific analyses at each timepoint/group.

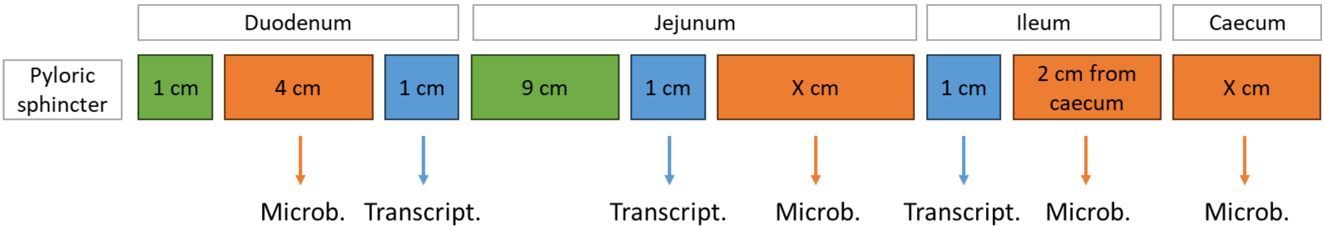

Figure S2. Sectioning of the mouse intestine for collecting digesta from duodenum, jejunum, ileum and caecum for microbiota analyses and tissue from duodenum, jejunum and ileum for transcriptomics analyses.

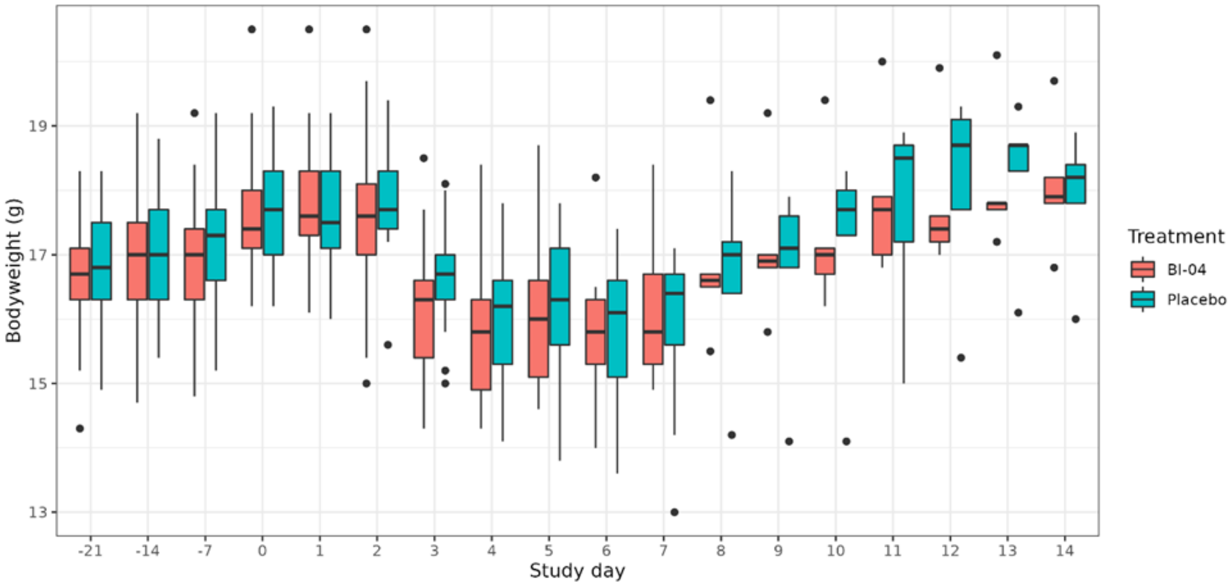

Figure S3. Mouse body weights over the course of the study. The middle line of each box represents the median of the data, the upper and lower hinges denote the first and third quartiles of the data. The whiskers extend to the lowest and highest value of the data, within 1.5 * the inter-quartile range, points that fall outside that range are shown as a dot. See Supplementary Figure 1 for n of mice at each timepoint.

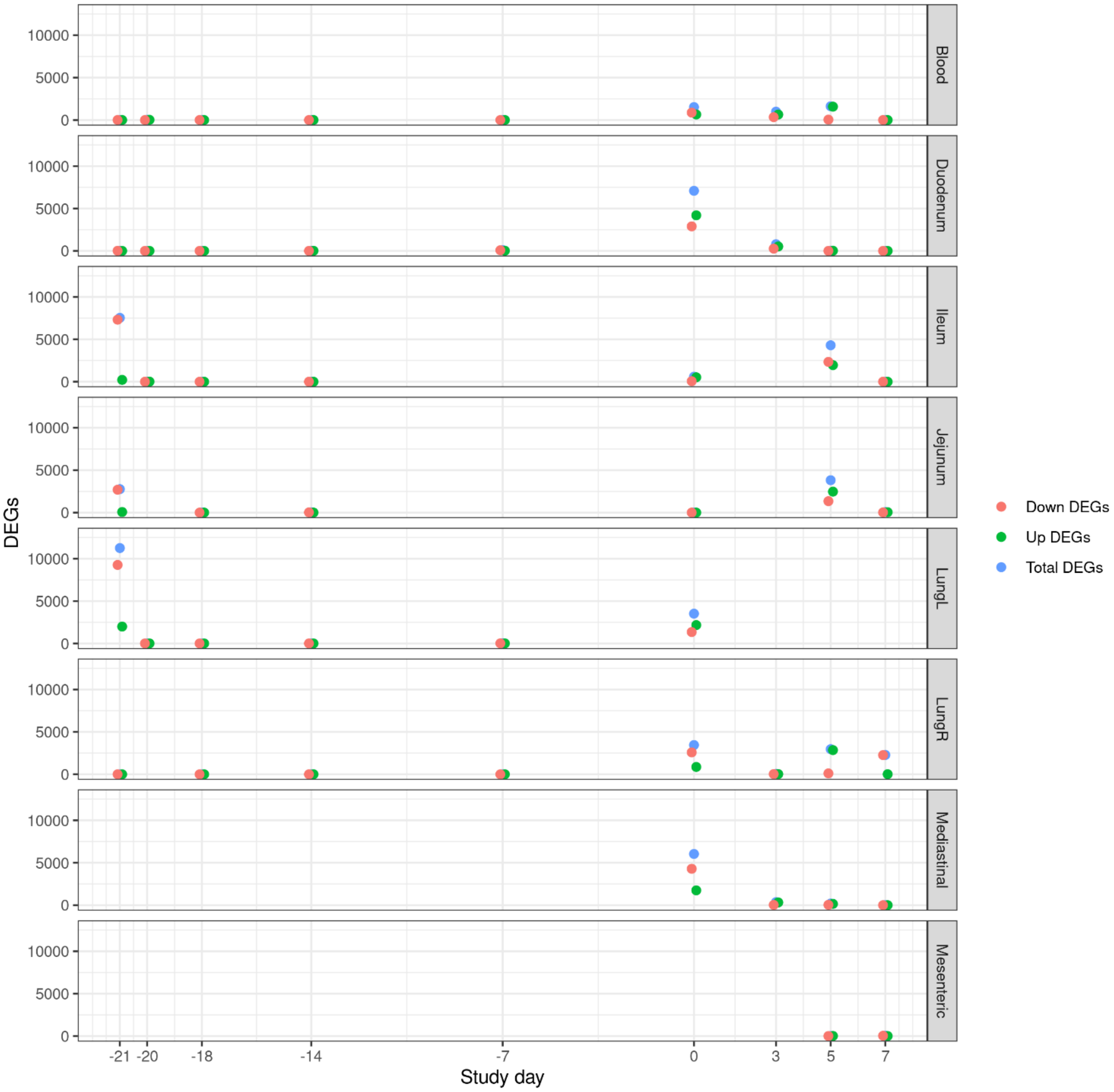

Figure S4. Differentially expressed genes (DEGs) in different mouse tissues in Bl-04 group compared to placebo group at each timepoint. DEG point chart showing the total number of DEGs in blue, upregulated DEGs in green, and down-regulated DEGs in red. There were missing data for some timepoints (Jejunum, LungL, Mediastinal, Mesenteric), with those timepoints missing from representation in the figure. LungL; left lung, LungR; right lung.

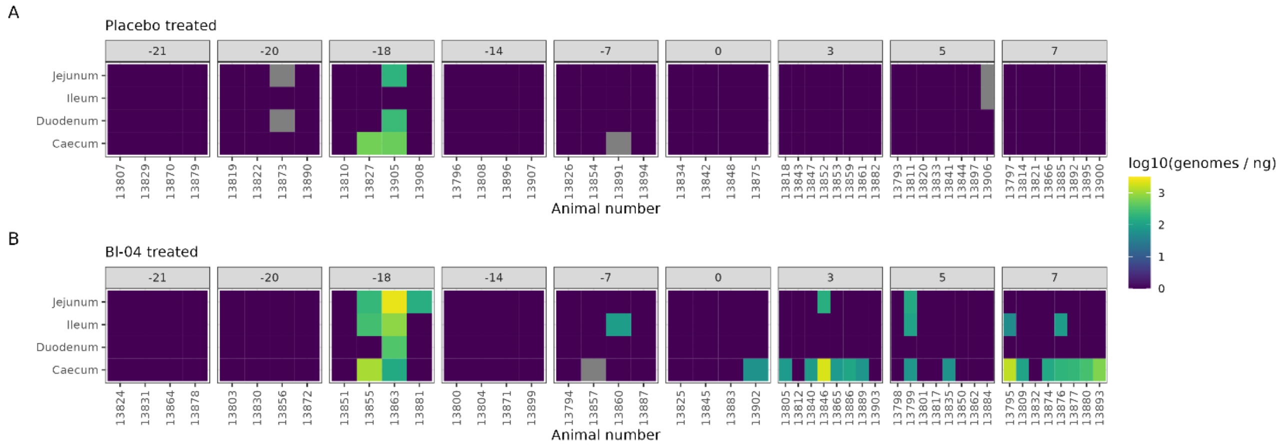

Figure S5. Heatmap showing qPCR estimates of Bl-04 genomes / nanogram of digesta from different tissues of placebo treated (a) and Bl-04 treated (b) mice. Animal number corresponds to an individual mouse sacrificed at a specific timepoint. Missing data was shown as a grey tile, whereas 0 values represent samples that were below the detection limit of the Bl-04-specific qPCR.

Table S1. Table of mapping results from quantifying probes using salmon against the Biospyder mouse probeset. Animal number corresponds to the individual mouse sacrificed to provide tissue samples, "Reads mapped" is the number of reads that mapped to the probe sequences, "Reads processed" is the total number of reads processed for that sample, and "Percent mapped" is the percent of the reads that mapped of the reads that were processed.

Table S2. Filtered DEG table - Excel file

Table S3. Filtered Pathway table – Excel file

Table S4. Alpha diversity (Faith's Phylogenetic Diversity PD metric) comparisons between placebo and Bl-04 study groups.

**Intestinal site**	**Group 1: Placebo**		**Group 2: Bl-04**		**P-value**[a]
		Faith's PD		Faith's PD	
	N	Mean ± SD	N	Mean ± SD	
Duodenum	19	11.57 ± 5.1	21	10.79 ± 4.7	0.61
Jejunum	33	10.47 ± 3.9	36	10.12 ± 4.9	0.49
Ileum	46	12.39 ± 4.5	44	10.80 ± 5.0	0.08
Caecum	46	19.78 ± 3.1	46	20.47 ± 2.3	0.10

[a]Placebo vs. Bl-04; Mann-Whitney U test.

Table S5. PERMANOVA (adonis) test describing the effect of study factors on beta diversity sample clustering from intestinal microbiota samples.

	**PERMANOVA**[a]	
**Factor**	**R^2**	**P-value**
Duodenum		
Study group (Placebo vs. Bl-04)	0.01	0.66
Time point	0.49	**0.001**
Study group*Time point	0.06	0.91
Jejunum		
Study group (Placebo vs. Bl-04)	0.01	0.31
Time point	0.31	**0.001**
Study group*Time point	0.10	0.31
Ileum		
Study group (Placebo vs. Bl-04)	0.020	0.072
Time point	0.23	**0.001**
Study group*Time point	0.07	0.53
Caecum		
Study group (Placebo vs. Bl-04)	0.02	**0.05**
Time point	0.29	**0.001**
Study group*Time point	0.01	0.49

[a]The PERMANOVA R^2 value represents the fraction of total variation of PC1 calculated from the weighted UniFrac pairwise sample distances (principal coordinates analysis; PCoA) that is attributed to a study factor, where the sum of all factors totals to 1.

Table S6. Excel data file containing microbiota abundance profiles for all samples.
